# Handheld ultrasound to avert maternal and neonatal deaths in 2 regions of the Philippines: an iBuntis® intervention study

**DOI:** 10.1186/s12884-018-1658-8

**Published:** 2018-01-18

**Authors:** Godofreda V. Dalmacion, Ramon T. Reyles, Antonia E. Habana, Lalaine Mae V. Cruz, Maricelle C. Chua, Analyn T. Ngo, Milagros J. Tia-Jocson, Emmanuel S. Baja

**Affiliations:** 1 Department of Pharmacology and Toxicology, College of Medicine, University of the Philippines-Manila, Manila, Philippines; 2 Department of Clinical Epidemiology, College of Medicine, University of the Philippines- Manila, Manila, Philippines; 3 Institute of Clinical Epidemiology, National Institutes of Health, University of the Philippines- Manila, Manila, Philippines; 4 Department of Obstetrics and Gynecology, Makati Medical Center, Makati, Philippines; 5 Department of Obstetrics and Gynecology, College of Medicine, University of the Philippines- Manila, Manila, Philippines; 6 Department of Obstetrics and Gynecology, Manila Doctors Hospital, Manila, Philippines; 7 Department of Obstetrics and Gynecology, College of Medicine, Manila Central University FDT Medical Foundation, Caloocan, Philippines

**Keywords:** Handheld ultrasound, Screening, Access to prenatal care, Maternal and neonatal mortality

## Abstract

**Background:**

The major causes of maternal and neonatal mortality in the Philippines are hemorrhages and obstructed labor due to placental implantation abnormalities (PIAs), twin pregnancies and fetal malpresentations. All of which are all easily detected by ultrasound. However, women in rural areas and low-income groups do not have access to ultrasound during their prenatal care. We aimed to provide additional evidence on the benefits of handheld ultrasound (HU) for screening pregnancy related abnormalities in order to avert maternal and neonatal deaths.

**Methods:**

Using a HU, we trained community healthcare workers (CHWs) to identify 5 obstetrical conditions: fetal viability and number, placental localization, amniotic fluid volume (AFV) and fetal presentation. Women, between 20th and 24th weeks age of gestation from 2 regions of the Philippines, were scanned using the HU and the GE Logic 5 Premium ultrasound machine for validation. Maternal and neonatal deaths averted were estimated as health outcome measures of the study.

**Results:**

Four hundred sixty women were scanned of which 146 (31.7%) showed abnormal ultrasound readings consisting of 17 PIAs, 123 fetal malpresentation, 3 twins and 3 AFV abnormalities. The use of HU could have possibly averted 29 (6.3%) maternal deaths and 14.6% neonatal deaths at the time of delivery. Thirty-two out of the 460 women (~7%) delivered at home and 93% in hospitals or birthing facilities/lying–in centers. We observed approximately 95% agreement between the ultrasound readings of the trainees and the trainers, and 99% agreement between the readings made from the HU with the validation machine.

**Conclusion:**

CHWs could be trained in the use of HU for scanning 5 obstetrical parameters. Early detection of abnormalities in these 5 obstetrical parameters can lead to early referral to facilities that are better equipped to manage obstetrical emergencies. Prenatal ultrasound can be an excellent point of care test for screening pregnant women at risk for possible complications and even death during labor and delivery.

**Trial registration:**

Thai Clinical Trial Registry identification number TCTR20171128004, retrospectively registered November 28, 2017.

## Background

Based on a 2013 UNICEF Philippine Report, 13 mothers die every day in the country from pregnancy-related complications [1]. It reported a high maternal death rate of 162 per 100,000 livebirths in 2000 escalating further to 221/100,000 livebirths in 2011 [1]. Neverthleless, there was a dramatic decrease in June 2015 to 115/100,000 livebirths which was still short of the 2015 Millennium Development Goal (MDG) of 15 per 100,000 livebirths. In 2015, the Philippines ranked 74th among 184 countries for maternal mortality rate (MMR) [2]. The major cause of maternal death in the country has been hemorrhage at 41% [1]. The different causes of maternal deaths worldwide have been attributed to hemorrhage (27%) of which 17% occurred in the postpartum period, sepsis (10.7%), obstructed labor (2.8%), hypertensive disorders (14%), and complications of unsafe abortion (7.9%). Most of these disorders are preventable with early diagnosis and prompt interventions [3]. Placental implantation abnormalities (PIAs) namely, placenta previa, marginal/low lying placenta, placenta accreta vasa previa and velamentous cord insertion account for both intrapartum and postpartum maternal hemorrhages [4]. They are identified by ultrasound, which normally can be done anytime between 18 and 22 weeks [4]. In contrast, the trend for neonatal mortality rate (NMR) of the Philippines or deaths in the newborn before they reach 28 days of life showed a downward trend from 20 per 1000 livebirths in 1990 to 12.6 per 1000 livebirths in 2015. This was comparable to Indonesia’s estimated 13.5 neonatal deaths per 1000 livebirths [5]. The top three causes of newborn deaths in the Philippines are prematurity (31%) followed by asphyxia and birth trauma (23%) and congenital anomalies (19%), which are congruent with the worldwide statistics [6]. Nevertheless, PIAs contributed substantially to preterm birth with prematurity rates ranging from 38 to 82% simply because premature termination of pregnancy in many instances may be the definitive management for PIAs [7]. Several studies on maternal health traced the link between neonatal and maternal deaths to the lack of adequate health care during pregnancy [3]. Poor access to obstetric ultrasound in rural areas of developing countries is known to be significantly detrimental to perinatal outcomes [8]. A separate study in 2011 reported not only the under-ultilization of ultrasound imaging in many developing countries but also the lack of trained sonographers as a challenge in delivering quality maternal healthcare [9].

In the Philippines, even the cheapest handheld types are not available in the government birthing facilities. In addition, ultrasound machines can only be found in government Tertiary Hospitals where most Obstetrician (OB)-Sonographers practice. In primary and secondary health facilities, the prenatal management is confined to measuring the vital signs of the pregnant patient, performing physical examination including the Leopold’s Maneuver and doing routine blood tests. A study in Botswana, involving 2309 patients, who attended an Ultrasound Service in a district hospital showed fetal demise, spontaneous abortion, low lying placenta and ectopic pregnancy as among the obstetrical pathologies. It found out that offering ultrasound services during the prenatal visits are cost effective and can improve patient care by 30% [10].

Active surveillance of the pregnant patient with abnormal obstetrical images on ultasound can be initiated earlier, reducing the development of conditions to a more catastrophic event. Unnecessary internal examination, which can potentially result to undue vaginal bleeding, can also be avoided by the early identification of abnormal placental implantation. Lastly, adequate preparation such as improving the hemodynamic state of the parturient to withstand heavy blood loss during labor and delivery especially by cesarean section can be appropriately instituted avoiding possible blood transfusions during labor and delivery. A study of Oyelese and colleagues reported 97% (59/61) of infants prenatally diagnosed by ultrasound with vasa previa surviving compared with only 44% (41/94) survivors out of those undiagnosed [11]. Finally, Harris and Marks in 2009 summarized the anecdotal experience of ultrasound in low resource setting. They observed improvement of public health care including maternal care with the deployment of donated ultrasound units. They particularly cited the experience of a Nicaraguan physician who reported a reduction of maternal mortality from 12 deaths per year to 5 per year in his practice. The study recommended doing field trials using ultrasound against a reference validation machine on a scale equal with public health interventions [12].

The objectives of the study were: (1) to ascertain the benefits of early ultrasound imaging of pregnant women in their 20th to 24th weeks age of gestation in detecting PIAs, fetal conditions such as malpresentations and multiple pregnancies; (2) to estimate the agreement between the ultrasound readings of the trainees with their trainers; (3) to validate the readings from the handheld ultrasound (HU) with the reference ultrasound validation machine; (4) to determine the type of birth attendant and place of deliveries of our study population representing both rural and urban sites and (5) to estimate the maternal and neonatal deaths possibly averted from the use of a HU in their prenatal care.

## Methods

### Study sites and population

Parañaque city, the urban study site and Tagum city, the rural study site, are located in 2 different regions of the Philippines. They were purposively selected as study sites based on their similar MMR of 1 per 1000 livebirths. Parañaque consisting of 17 barangays with a population of 582,000 is a highly urbanized city located in the National Capital Region. Tagum, located in the province of Davao del Norte is an agricultural city rich in mineral deposits, has 23 barangays and a population of 242,000. The study participants were randomly sampled from the list of pregnant women in their 20th to 24th weeks of pregnancies during which time placental localization is more reliably established [5]. Before the 18th weeks age of gestation, 50% of placenta will be low lying and between 18th and 24th weeks age of gestation it is estimated that 2–4% will still remain low lying. Thus, the Society of Obstetrician and Gynecologists of Canada recommends repeating the ultrasound for PIAs by the third trimester to confirm the final localization of the placenta [13]. All the towns locally known as barangays from each study site were included in the sampling frame. The number of samples from each town was proportional to the size of its population. Excluded were pregnant women with known allergy to gel, with concurrent medical or surgical conditions and those who were not able to provide informed consents. Prior to the implementation of the project, the study proposal was reviewed and approved by the National Ethics Committee. The informed consent was written in the local dialect and was administered to all women who were recruited. Women who signed the informed consent were eligible and included in the study.

### Study design and sample size

A cross sectional study design was used. The sample size was calculated based on the prevalence of abnormal ultrasound images of 35% in the second trimester of pregnancy reported from an earlier study [14].

### Ultrasound technical working group

All cities and municipalities in the country have a community healthcare team (CHT), which is composed of general practitioners, nurses, and midwives. A technical working group (TWG) composed of 10 members of the CHT was identified for each study site to act as coordinators and to undergo training in ultrasound scanning. A total of 20 ultrasound trainees, consisting of 6 doctors, 5 nurses, and 9 midwives from both cities, were selected by their respective City Health Officer and subsequently trained in scanning. The training package was composed of classroom lecture, hands-on training and 2–3 days of actual scanning of the pregnant women recruited by the study. The Training Team administered a pre-test prior to the training proper to establish the baseline knowledge of the selected TWG-trainees on obstetrical ultrasound. All the lectures were delivered by the head of the scanning team who was then the President of the Philippine Society of Ultrasound in Obstetrics and Gynecology (PSUOG). All trainees were taught on how to scan using a mobile handheld General Electric (GE) ultrasound called VScan. The GE VScan has a dual probe that can be used to scan the pelvic and abdominal organs like the liver and the heart. It comes with a docking station and 4 GB micro-SD memory card. The VScan does not have a M-Mode and therefore cannot give an actual count of the fetal heart rate.

The TWG were trained to identify and measure 5 obstetrical ultrasound images namely; viability of the fetus, number of pregnancy, fetal presentation, location of the placenta and amniotic fluid volume (AFV) using maximal vertical pocket. By focusing only on specific obstetrical parameters, we were able to effectively train our community healthcare workers (CHWs) for a short period of time and avoided any possible conflicts with the practice of the credentialed OB–sonographers of the site. After the hands-on training, a written post-test was administered to the TWG-trainees to determine improvement of their knowledge on the principles of ultrasound imaging from baseline.

### Ultrasound iBuntis screening intervention

Streamers and banners with the iBuntis Logo were posted in the different health facilities to effectively recruit pregnant volunteers. The demographic and obstetrical data of the pregnant recruits who fulfilled the inclusionary criteria were collected even prior to the free ultrasound sessions which were conducted for three days in urban study site and two days in the rural study site. During the actual ultrasound sessions, the TWG-trainees of each study city performed the initial ultrasound scan using the VScan. Their ultrasound readings were instantly confirmed by the trainers using the same VScan and both their readings further confirmed by the head sonologist of the study using the GE Logic 5 Premium ultrasound as the reference standard. Thus, sources of the disagreements and errors of the trainees were immediately identified, explained and corrected by the trainers. At the end of the study, each trainee on average scanned 30 women.

Patients with abnormal readings were immediately tagged and referred for more intensive prenatal surveillance under an accredited birthing facility or an Emergency Maternal and Neonatal Care unit. The ultrasound results were shared with the participants who were advised to repeat their ultrasound by the 32nd weeks age of gestation. Thereafter, midwives were commissioned to follow up all women for their pregnancy outcomes.

### Health outcome measurements

For the purpose of estimating the health outcome, we classified the different abnormal scans into those which can potentially lead to maternal deaths and those which are more likely to cause neonatal deaths. All abnormal ultrasound findings can actually lead to catastrophic events in either mother or neonate or both. Nevertheless, by adopting a specific operational definition of “deaths averted” based on the ultrasound results we can calculate our health outcomes. Thus possible “maternal deaths averted” were estimated as percent possible cases of deaths from PIAs and transverse lie only over the total number of scans, while possible “neonatal deaths averted” were estimated as percent cases of twin, breech presentation and AFV abnormalities over the total number of scans. We likewise assumed that even if abnormal ultrasound images can eventually become normal at birth e.g. breech to cephalic and low-lying to normal placentation, possible catastrophic perinatal outcomes were considered averted by their timely referral to better-equipped hospitals and more targeted prenatal surveillance.

### Data collection and management

Using the case report forms (CRF) of the study, data were extracted in the CRF, double encoded into a Google form version of the CRF, and matched for any data entry discrepancies. Any inconsistencies in the Google database were resolved by cross-checking with the CRFs. Access to the Google database was limited to the Principal Investigator and the biostatistician/epidemiologist of the study.

### Statistical analysis

We calculated descriptive statistics for all the health outcomes and covariates of the study population. Mean and the standard deviation were used to summarize continuous variables while frequencies and the percentages were used to summarize categorical variables. Association between the study sites (Tagum and Parañaque) and the categorical health outcomes were assessed using either Fisher’s Exact test or Pearson’s Chi-square test and using Student’s t-test for continuous variables. *P*-values ≤ 0.05 were considered statistically significant. STATA v13 (StataCorp, College Station, TX, USA) was used in the analysis.

## Results

A total of 462 pregnant women were recruited but only 460 met the inclusionary criteria. Two hundred fifty pregnant women were recruited from the urban Paranaque study site and 210 from the rural Tagum study site. The characteristics of the pregnant women are summarized in Table 1. The women in Paranaque were slightly older than the women in Tagum (mean age in years ± standard deviation (SD): 26.0 ± 6.4 vs. 25.0 ± 6.2, *p*-value = 0.09), and 1 out of 10 women had irregular menstrual cycles for both study sites. Although the duration of training in the urban site took longer than in the rural site, there was no difference in the scores of the pre- and post- test of the trainees or their performances in the actual scanning sessions for both rural and urban sites. A total of 20 CHW trainees with 78 hours of training was produced and 3 training modules were created.

**Table 1:** Characteristics of study population from two regions in the Philippines (*N* = 460)

Characteristic	Rural Tagum (*n* = 210)	Urban Parañaque (*n* = 250)	*p*-value
Age (years), *mean* [SD]^a^	25.0 [6.2]	26.0 [6.4]	0.09
Age of 1st menstruation (years), *mean* [SD]	13.1 [1.6]	13.4 [1.7]	0.053
Smoking status, *N* (%)			
Ever	31 (14.8)	36 (14.4)	0.91
Never	179 (85.2)	214 (85.6)	
Contraceptive usage, *N* (%)			
Ever	108 (51.4)	121 (48.4)	0.52
Never	102 (48.6)	129 (51.6)	
Menstrual cycle, *N* (%)^b^			
Irregular	22 (10.5)	27 (10.8)	0.91
Normal	188 (89.5)	223 (89.2)	
Had a pap smear procedure, *N* (%)			
Yes	31 (15.7)	34 (13.6)	0.55
No	166 (84.3)	214 (85.6)	
Missing	13		

^a^*SD* Standard deviation; ^b^Irregular menstrual cycle: < 28 days or >35 days

Table 2 shows the possible maternal and neonatal deaths averted in both study sites. One hundred forty-six of the 460 pregnant volunteers (31.7%) had abnormal scans. There was a statistically significant difference in the number of abnormal scans between the urban Parañaque study site and the rural Tagum study site with the former having a higher prevalence of abnormal ultrasound results (37.6% vs. 24.8%, *p*-value <0.01). The distribution of abnormal scans included abnormal AFV (0.6%), twin pregnancy (1.3%), PIAs (3.7%) and fetal malpresentation (26.7%). The most common fetal malpresentation was breech (24.1%) followed by transverse lie (2.6%). The total maternal and neonatal deaths possibly averted at the time of the scanning from both study sites were estimated to be 6.3% and 26.1%, respectively using the predetermined definition. Furthermore, higher percentages of deaths possibly averted were observed in urban Parañaque compared to rural Tagum for both maternal (8.8% vs. 3.4%, *p*-value = 0.02) and neonatal (30% vs. 21.4%, *p*-value = 0.02) deaths. At the time of delivery, 40% of the 111 breech presentations turned to cephalic which decreased the neonatal deaths possibly averted to approximately 14.6% (67/460).

**Table 2:** Maternal deaths and neonatal deaths possibly averted in Parañaque City and Tagum City

Health Outcome	Total (*N* = 460)	Urban Parañaque (*N* = 250)	Rural Tagum (*N* = 210)	*p*-value
Ultrasound findings (%)				
Normal scans	68.3	62.4	75.2	< 0.01
Abnormal scans	31.7	37.6	24.8	
Maternal deaths possibly averted^a^ (%)				
Normal	93.7	91.2	96.6	0.02
Abnormal placentation				
Low lying	2.4	2.4	2.4	
Placenta previa	1.3	2.0	0.5	
Fetal malpresentation				
Transverse	2.6	4.4	0.5	
Neonatal deaths possibly averted^b^ (%)				
Normal	74.6	71.2	78.6	0.02
Fetal malpresentation				
Breech	24.1	26.8	20.9	
Oligohydramnios	0.2	0.0	0.5	
Polyhydramnios	0.4	0.8	0.0	
Twins^c^	1.3	2.4	0.0	

^a^maternal deaths averted = maternal deaths due to bleeding from abnormal placental localization and transverse lie; ^b^neonatal deaths averted = neonatal deaths due to fetal malpresentation; ^c^neonatal deaths averted for twins = actual number of twins × 2

Table 3 summarizes the distributions of the referral to health facility and the place of delivery of the study participants. There was no difference between the two study sites regarding home deliveries of women with abnormal scans (*p*-value = 0.70). However, more women with abnormal findings in the urban study site delivered in hospitals (24.4% vs 14.3%, *p*-value <0.05), and in birthing facilities/lying in (11.6% vs. 8.1%, *p*-value = 0.02), than those residing in the rural study site.

**Table 3:** Health facility referral and place of delivery based on study site

Outcome parameter	Total (*N* = 460)	Urban Parañaque (*N* = 250)	Rural Tagum (*N* = 210)	*p*-value
Health facility referral (%)				
Hospital	52.4	56.8	47.1	0.11
Birthing facility / Lying in	40.6	36.4	45.7	
Home	7.0	6.8	7.2	
Place of delivery (%)				
Hospital				
Normal cases	32.6	32.4	32.9	0.046
Abnormal cases	19.8	24.4	14.3	
Birthing facility / Lying in				
Normal cases	30.7	24.8	37.6	0.02
Abnormal cases	10.0	11.6	8.1	
Home				
Normal cases	5.0	5.2	4.8	0.70
Abnormal cases	2.0	1.6	2.4	

Doctors from the two study sites attended 19.7% of the abnormal birth deliveries followed by midwives (10.0%), and traditional birth attendants (TBA) at 2.0% (Table 4). More pregnancies with abnormal scans were delivered by Physicians in the urban site compared to those in the rural site (24.4% vs. 14.3%, *p*-value = 0.03). Conversely, more pregnancies with normal scans were delivered by midwives in the rural site compared to the urban study site (36.2% vs. 24.8%, *p*-value = 0.03). In addition, neonatal mortality, still birth and congenital anomalies were only observed in the rural study site with prevalence of approximately 10 per 1000 livebirths, 5 per 1000 livebirths and 10 per 1000 livebirths, respectively.

**Table 4:** Types of birth attendants according to study site

Outcome parameter	Total (*N* = 460)	Urban Parañaque (*N* = 250)	Rural Tagum (*N* = 210)	*p*-value
Obstetrician (%)				
Normal cases	33.3	32.4	34.3	0.03
Abnormal cases	19.7	24.4	14.3	
Total	53.0	56.8	48.6	
Midwife (%)				
Normal cases	30.0	24.8	36.2	0.03
Abnormal cases	10.0	11.6	8.1	
Total	40.0	36.4	44.3	
Traditional birth attendant (%)				
Normal cases	5.0	5.2	4.8	0.70
Abnormal cases	2.0	1.6	2.4	
Total	7.0	6.8	7.2	

Very good agreement at 95% was observed between the ultrasound readings of the trainee CHWs with those of the credentialed trainers. Furthermore, excellent agreement at 95.7% was also observed between the readings made from the HU and the reference machine. Most of the disagreements were observed on fetal presentation. The trainess-CHWs tend to misdiagnose breech and transverse lie as cephalic (see Table 5 for details).

**Table 5:** Agreement between the handheld ultrasound and the reference bigger ultrasound machine for selected Obstetrical ultrasound parameters (*N* = 460)^a^

Ultrasound parameter	In agreement [N, (%)]	Not in agreement [N, (%)]
Fetal presentation	440 (95.7%)	20 (4.3%)^b^
Amniotic fluid	458 (99.6%)	2 (0.4%)
Location	459 (99.8%)	1 (0.2%)
Fetal number	460 (100.0%)	0 (0.0%)

^a^Handheld ultrasound: GE VScan and Reference standard ultrasound: GE Logic 5 Premium

^b^Not in agreement breakdown: Cephalic-Breech (9), Cephalic-Transverse (4), Breech-Transverse (3), Transverse-Breech (2), and Breech-Cephalic (2)

The cost for the intervention in both study sites was 1.7 million pesos (PhP 1.7 M), with a higher cost of the intervention in the rural study site (PhP 981,000.00) compared to the urban study site (PhP 719,000.00). The PhP 262,000.00 difference in cost was due to the air fares spent to conduct the study in Tagum. The capital outlay for the equipment was reasonably priced at PhP 350,000.00 for each study site (see Table 6 for details).

**Table 6:** Cost of intervention for the two study sites

Outcome parameters	Total	Urban Parañaque	Rural Tagum
Cost in PhP			
Equipment	700,000.00	350,000.00	350,000.00
Training	570,000.00	285,000.00	285,000.00
Travel	292,000.00	15,000.00	277,000.00
Consultants	138,000.00	69,000.00	69,000.00
Total	1,700,000.00	719,000.00	981,000.00

## Discussion

Access to prenatal care and services by pregnant women in the Philippines has been restricted mainly due to the following reasons; limitations of diagnostic equipment and treatment modalities in the health facilities, the nature of its geography being an archipelago with bodies of water separating hospitals from their clients, cost of health care and the lack of maternal health advocacy. The project was named iBuntis, a local and short term for “Information Pregnancy”. We observed that almost all abnormal ultrasound findings seen between the 20th and 24th weeks age of gestation have not given rise to any clinical signs and symptoms that necessitated emergency referrals to a specialist or hospital. But with the ultrasound scanning done earlier, abnormal findings were shared with the City Health Officers of both study sites. This allowed the tagging of all the pregnant participants at risk for complications during labor and delivery to allow closer surveillance. These referrals could have helped pregnant women since there were no maternal deaths reported from both study sites.

The results of our study showed that screening of high-risk pregnancies could provide more targeted follow-up and management of pregnancies. It is worth mentioning that only 42.6% (40/94) of patients with abnormal scans in the urban study site and none in the rural study site went for the repeat scan. We hypothesize the reason for failing to do so was the lack of funds to pay for the repeat ultrasound. In spite of the issuance of an administrative order that prohibited home birth delivery prior to the conduct of the iBuntis study, 6.2% (9/146) of the abnormal cases still delivered at home. The observed 6.2%, however, is lower than the reported 9.1% of births attended at home by TBA in 2015 [15]. Nevertheless, more effective information campaigns on the benefits of hospital and birthing facility delivery could be requested from the Department of Health and the local governments. Furthermore, 93.8% (137/146) of the abnormal scans delivered at either the referral hospitals or birthing facilities. We could attribute the effective high referral outcome to women going with additional information in the form of an ultrasound reading about their conditions.

Our study successfully showed that all CHWs from doctors, nurses to midwives are highly trainable and competent in using the HU. Capacitating CHWs to perform screening ultrasound for 5 obstetrical parameters is highly tenable. Brunette et al. reported a similar finding among midwives in Uganda [16]. Midwives were successfully trained on the use of a low-cost, portable and easy-to-use ultrasound. As a consequence of their training, they were able to effectively identify high risk conditions for referrals to bigger hospitals and birthing facilities. With the advancing technology for smaller and mobile ultrasound machines and their lower cost, our local governments may eventually be able to procure handheld ultrasounds as part of their armamentarium for good prenatal care. Nevertheless, more studies in the cost-effectiveness of ultrasounds in rural and urban settings are still needed. Interestingly, we learned that ultrasound screening could influence the decision to use postpartum family planning methods. About 30–35% of women who were scanned asked for the sex of their unborn child and articulated their intention to practice postpartum contraception, or even surgical sterilization, if the child they are carrying carries the sex that they or their husbands wish for. In our study, we also learned that HU should not be used to scan for congenital defects. With a focused ultrasound screening using a 3.5-in. monitor, congenital anomalies could be easily missed. A more extensive training in obstetrical imaging is required for one to develop the ability to detect congenital anomalies using the bigger ultrasound. Based on the Birth Defects Surveillance of the Philippines of 2011, congenital anomalies rank third in the list of common causes of death in infancy in the country [17]. In our study, all congenital anomalies were observed in the rural site, Tagum. A more comprehensive environmental exposure assessment study among pregnant women should be done in similar rural study sites.

Our study showed that the handheld ultrasound at the “point of care” might successfully screen for abnormal obstetrical conditions, which can potentially lead to maternal and neonatal deaths or complications. Using surrogate indicators, 6.3% of maternal deaths and 14.6% of neonatal deaths were possibly averted by the early ultrasound screening. We also observed that socio-cultural beliefs could be stronger determinants than knowledge and logical reasoning for making decision on childbirth and labor. This was manifested by one of our patients who was diagnosed to have a fetus in breech presentation but refused to deliver in the hospital despite warnings that she and her child were at risk for complications and birth injuries respectively. We recommend more studies on the psyche, beliefs and socio-cultural factors affecting women to have a safe labor and delivery.

One limitation of the study is the limited number of study sites due to restricted funding. However, our study design enabled us to explore the contrast between pregnant women living in an urban setting vs. a rural setting. A more expanded ultrasound intervention study is warranted to verify the results of our project. We recommend a scale up study to other regions especially to geographically isolated areas of the country through their Local Government Officials. We also recommend doing the screening in a larger number of pregnant women against a control group without the procedure to estimate its cost effectiveness in averting possible maternal and neonatal deaths.

## Conclusion

Very few pregnant women in the rural areas of low to low-middle income countries actually have any information about the location of their placentas and the conditions of their fetuses in the prenatal period. Absence of information lowers the chance of pregnant women avoiding catastrophic events during their labor and delivery. General practitioners, nurses and midwives were highly trainable and reliable in performing ultrasound scanning for five obstetrical parameters namely, fetal number and viability, fetal presentations, amniotic fluid volume and placental localization. Identification of these conditions can possibly avert maternal deaths and neonatal deaths. Lastly, the Local Government Units and the CHWs are key players in improving pregnancy outcomes in the country.

## Abbreviations

AFV: Amniotic fluid volume
CHT: Community Health Team
CHW: Community Health Worker
GE: General Electric
HU: Handheld Ultrasound
LGU: Local Government Unit
MMR: Maternal Mortality Rate/Ratio
OB: Obstetrician
TBA: Traditional Birth Attendant
TWG: Technical Working Group
